# The Quality of Online Information for the Treatment of Knee Osteoarthritis: A Google Study

**DOI:** 10.7759/cureus.29995

**Published:** 2022-10-06

**Authors:** Breanna Sullivan, Varag Abed, Josh Joiner, Max Benningfield, David Landy, Gregory S Hawk, Caitlin Conley, Cale Jacobs, Austin V Stone

**Affiliations:** 1 Orthopedic Surgery, University of Kentucky, Lexington, USA; 2 Statistics, University of Kentucky, Lexington, USA

**Keywords:** osteoarthritis, online, aging, google, oa

## Abstract

Introduction

Affecting more than 30 million adults annually, osteoarthritis (OA) is the most common joint disorder in the United States. A variety of management options for knee OA exists, including physical therapy, weight loss, intra-articular corticosteroid injections, and total joint arthroplasty. With many treatments available, patients often utilize the internet to educate themselves about their condition and management options. The purpose of this study was to evaluate the quality, transparency, and readability of online information for the treatment of knee OA.

Methods

The search terms “knee,” “arthritis,” and “treatment” were entered into an incognito mode Google browser. Websites were classified by type (commercial, academic, nonacademic medical practice, government/patient advocacy, and other) and graded on content quality, transparency, and readability using the following scores, respectively: modified DISCERN, Journal of American Medical Association (JAMA) Benchmark, and Flesch-Kincaid (FK) grade level.

Results

Of the 95 websites evaluated, commercial (mean, 38.2) and academic (37.3) sites had the highest total DISCERN scores, which were significantly greater than nonacademic medical practice (31.8) and government/patient advocacy sites (33.4) (p≤0.035). Nonacademic medical practice sites had the lowest mean total DISCERN (31.8) and JAMA (1.47) scores and the highest FK grade level readability (9.5). There was a significant positive correlation between mean total DISCERN and JAMA scores (r=0.46, p<0.001).

Conclusion

The mean overall quality of websites regarding the treatment of OA was good as evidenced by greater modified DISCERN scores. However, website quality ranged from poor to excellent, indicating that some websites are still missing key information patients may find useful when assessing treatment options online.

## Introduction

Affecting more than 30 million adults annually, osteoarthritis (OA) is the most common joint disorder in the United States [1]. The median age at diagnosis of knee OA is 55 years [2], and it affects up to 10% of men and 13% of women older than 60 years [3]. As life expectancy and obesity increase, OA is becoming more prevalent [4]. A variety of management options for knee OA exists, including physical therapy, weight loss, intra-articular corticosteroid injections, and total joint arthroplasty [5]. Patient education is also a key component of OA management [6]. With many treatments available, patients often utilize the internet to educate themselves about their condition and management options [7-9]. There are about 6.5 million health-related Google searches per day [10], and Google is utilized in 75% of searches for health-related information [11]. Among orthopedic patients, 49% search their condition on the internet before their visit with a physician, and 42% search the internet following their visit, which emphasizes the importance of the internet as a tool for patient education [12].

A continued increase in Google searches for the term “osteoarthritis” over recent years may reflect the increasing prevalence of OA and the concomitant rise in internet usage among patients [11]. Although there has been an increase in availability and usage of the internet to learn more about OA, this does not necessarily translate to an increase in the quality of information available online. The types of websites patients can be directed to vary but include commercial, academic, nonacademic medical practice, government, and patient advocacy, among other websites such as news, social media or general information sites [13,14]. A 2005 study of the quality of online health information regarding OA diagnosis and treatment found that the overall quality of website information was poor, as measured by the DISCERN tool [15]. The DISCERN tool is a grading tool designed to help health consumers and healthcare professionals assess the quality of information regarding treatment options [16]. A more recent study investigating the readability and quality of online OA information found that overall website quality was “fair” [17], demonstrating a small improvement in the quality of information with time.

With the popularity of medical searches relating to knee OA, it is important to assess the quality of online information most frequently encountered in queries. The purposes of this study were to evaluate the overall quality of online information regarding treatment options for knee OA from frequently visited websites and to determine if website content quality correlates with website type. We hypothesized that commercial and academic websites would have higher quality scores than nonacademic medical practice, government/patient advocacy, and other websites. Additionally, we hypothesized that higher website quality would correlate with higher JAMA and readability scores.

## Materials and methods

The search terms “knee,” “arthritis,” and “treatment” were entered into an incognito mode Google browser. The incognito mode disables tracking data which could influence search results based on user activity or previous search histories [18]. Additionally, the more general search term “arthritis” was used instead of “osteoarthritis” because this term may be more familiar to the general population. After entering the search terms, a list of “People Also Ask” (PAA) questions is generated. This is a list of questions suggested by Google related to the original search query. This list of questions is based on what others have previously asked on Google, and thus represents frequently asked questions related to the original search query [19]. The first question in this list was clicked to refresh the list until 10 questions were generated (Table 1).

**Table 1:** Top 10 osteoarthritis questions

Questions
Can arthritis of the knee heal?
Is walking good for arthritis of the knee?
What are the five worst foods to eat if you have arthritis?
How should I sleep with arthritis in my knee?
How can I naturally lubricate my knees?
What helps arthritis in knee without surgery?
What are the first signs of arthritis in the knee?
Is it better to heat or ice a knee with arthritis?
What is the best painkiller for arthritis in the knee?
What exercises are bad for arthritic knees?

Next, each question was searched individually in an incognito mode Google browser, and the Uniform Resource Locators (URLs) of the first 10 websites suggested (excluding ads) were extracted to Excel. Three additional websites were extracted to account for two YouTube videos and one dysfunctional URL that were excluded. After review, five additional duplicate websites were excluded. Thus, a total of 95 websites were evaluated.

Two trained reviewers classified websites by type. Website categories were derived from established methodologies [13,14] and included: commercial, academic, and nonacademic medical practice, government/patient advocacy, and others. Definitions for these website classifications can be found in Table 2. Websites were then evaluated using the JAMA Benchmark Criteria, which consists of four items: authorship, attribution, currency, and disclosure. Websites are assigned one point for each item present, with the highest possible score being a four [20]. Because the JAMA Benchmark Criteria does not evaluate the content of the webpage, it was used as a measure of website transparency and verifiability.

**Table 2:** Description of website category *Single surgeon websites were categorized as a nonacademic medical practice. This does not necessarily reflect that the surgeon is not peripherally affiliated with an academic institution, but rather that they are being represented on their own since it is a personal website.

Website Category	Description
Commercial	Commercial organization that positions itself as a source of health information Example: Healthline.com, WedMD.com
Academic	Institution with clear academic mandate, including universities, academic hospitals and academic societies Example: Mayoclinic.org, orthoinfo.aaos.org
Nonacademic medical practice	Local hospital, private medical group, or single surgeon* unaffiliated with academic institution Example: Orthoatlanta.com, westchesterhealth.com
Government/patient advocacy	Websites ending in .gov or those maintained by national government or government organization; Websites maintained by non-for-profit organizations with the primary goal of patient education and advocacy Example: Arthritis.org, creakyjoints.org
Other	Websites not clearly fitting the above categories, mainly media and general information websites Example: oregonlive.com

The quality of the treatment information on each website was evaluated using a modified DISCERN tool. The DISCERN tool consists of 16 questions regarding bias, risks and benefits of treatment, relevance, and overall quality of information [16,21]. Each question traditionally has answer choices ranging from one to five: one=No, two/three=Partially, and four/five=Yes [21]. To increase the precision and accuracy of the partial category (previously scored 2-4), the DISCERN answer choices in this study were modified to range from one to three (one=no, two=partially, three=yes). Thus, possible total DISCERN scores ranged from 16 to 48, with higher scores representing greater information quality. Similar to other studies [22,23], using the total DISCERN score, quality scores were grouped into categories of Excellent (40-48), Good (34-39), Fair (28-33), Poor (22-27), and Very Poor (16-21).

Though each question generated in the search did not directly ask about treatment options, all webpages assessed mentioned treatment options, and the quality of this discussion was graded using the DISCERN tool. Because the more general term “arthritis” was searched, some web pages included information on rheumatoid arthritis (RA) in addition to OA. Since the focus of this study was knee OA, information regarding RA and its treatment options was not included in the website grading.

The grade level readability of websites was also assessed using the Flesch-Kincaid (FK) grade level score, which assigns an approximate reading grade level to the web page's text [24]. The FK score was obtained by using the Google Chrome plug-in Crafty Level [25]. The grade level assigned to a text indicates that a person who has completed that grade should be able to read and comprehend the information. Thus, a higher grade level indicates a more difficult text [26].

Statistical analysis

For each of the three website measures described above, summary statistics were calculated overall and broken out by website category. An overall Fisher’s Exact test was used to analyze differences in JAMA Benchmark score distribution across the five website categories listed above, while one-way ANOVA models were used to analyze the analogous differences in total DISCERN and FK readability scores. For measures in which the overall model was significant, relevant pairwise differences were calculated for the website categories. Additionally, a series of linear regression models and associated correlation analysis was used to examine the overall relationships among the three outcomes. Diagnostic measures and residual analysis were used to check model assumptions in each case. Throughout the study, a p-value of less than 0.05 was considered statistically significant. All analyses were completed in R, version 4.1.2 (R Foundation for Statistical Computing; Vienna, Austria).

## Results

**Figure 1:**
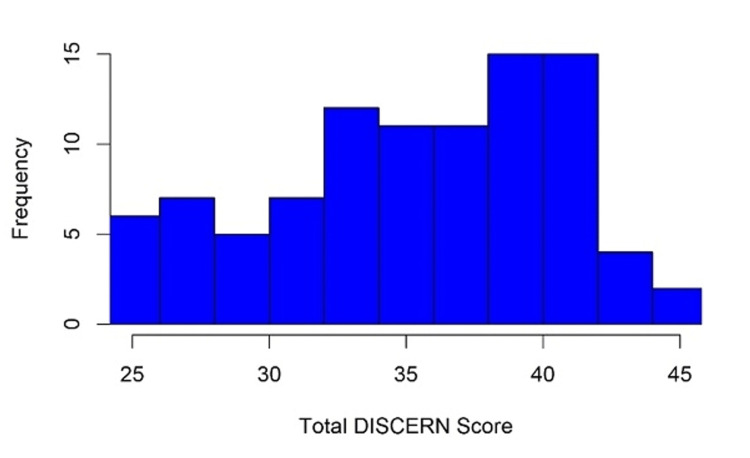
Histogram of total DISCERN score

A total of 95 websites were evaluated. The most common website types were commercial (46.3%) and nonacademic medical practice (20.0%). The mean overall JAMA Benchmark score was 2.24. The mean FK grade level readability of all websites was 8.3, and grade levels ranged from 3.4-11.8. The mean total DISCERN score was 35.8. Total DISCERN scores ranged from 24 to 45 (poor to excellent). The mean individual DISCERN score was 2.24 (Figure 1).

When evaluating individual website types, commercial (mean ± SD, 38.2 ± 4.5) and academic (37.3 ± 3.9) had the highest total DISCERN scores. Nonacademic medical practice websites had the lowest total DISCERN score (31.8 ± 4.8). The mean total DISCERN score of commercial websites was significantly greater than that of nonacademic medical practice (p<0.001) and government/patient advocacy websites (p=0.001). The mean total DISCERN score of academic websites was also significantly greater than nonacademic medical practice (0.002) and government/patient advocacy websites (0.035). Websites in the “other” category had the highest mean JAMA Benchmark score (3). However, it is notable that there were only four “other” websites. Following this, commercial (2.66) and academic (2.58) websites had the highest mean JAMA Benchmark scores. Nonacademic medical practices had the lowest mean JAMA Benchmark score (1.47). The mean JAMA Benchmark score of commercial websites was significantly higher than nonacademic medical practice (p<0.001) and government/patient advocacy websites (p<0.001). The mean JAMA Benchmark score of academic websites was also significantly higher than both nonacademic medical practice (0.0003) and government/patient advocacy sites (p=0.001). The highest mean FK grade level readability (9.5) was on nonacademic medical practice websites, while the lowest grade level (7.7) was found on government/patient advocacy sites. Nonacademic medical practice sites had a significantly higher FK grade level compared to commercial (p=0.003) and government/patient advocacy websites (p=0.004) (Table 3).

**Table 3:** Grading Criteria by Website Type FK: Flesch-Kincaid; JAMA: Journal of American Medical Association

Website Type (n)	Mean JAMA Benchmark (1-4)	Mean FK Grade Level	Mean Total DISCERN (16-48)
Commercial (44)	2.66	8.0	38.2
Academic (12)	2.58	8.5	37.3
Nonacademic medical practice (19)	1.47	9.5	31.8
Government/patient advocacy (16)	1.56	7.7	33.4
Other (4)	3.00	8.1	33.2

**Figure 2:**
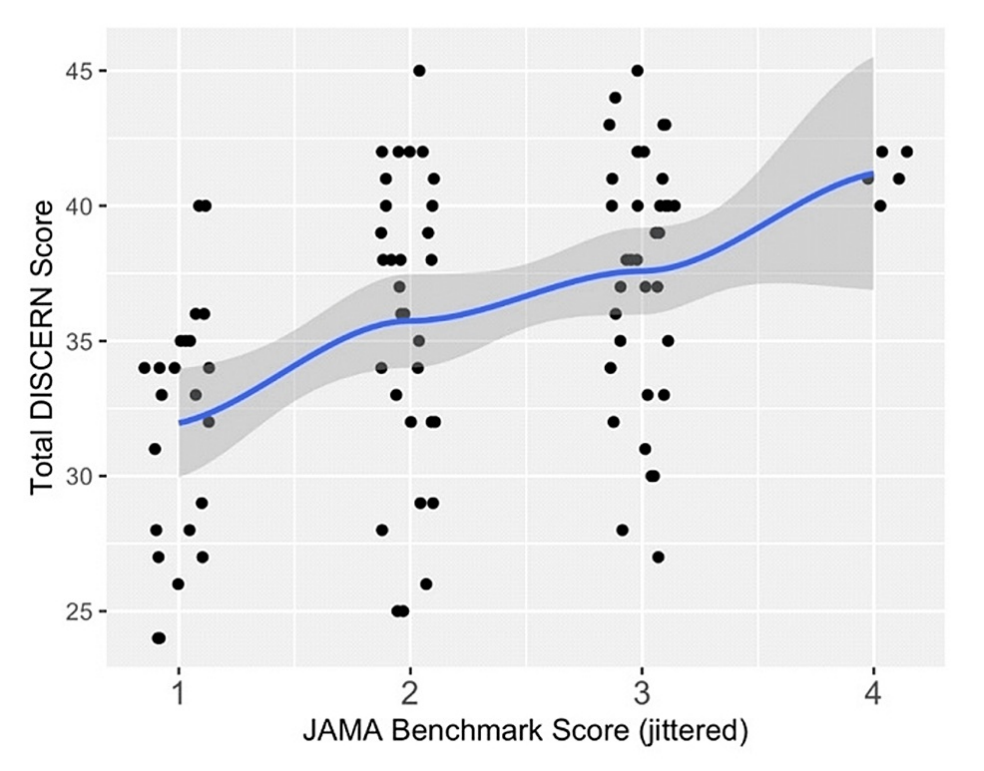
Mean total DISCERN score vs. JAMA benchmark score A LOESS-type smoother is overlaid in blue with a 95% confidence band in grey JAMA: Journal of American Medical Association

When evaluating the association between variables, there was no significant correlation between FK readability and JAMA benchmark scores (estimated Pearson correlation = -0.133; p= 0.200). There was also no significant correlation between total DISCERN and FK readability scores (estimated Pearson correlation = -0.061; p = 0.560). However, there was a significant correlation between total DISCERN and JAMA Benchmark scores (estimated Pearson correlation = 0.460, p< 0.001). For each additional JAMA Benchmark point increase, the estimated mean total DISCERN score increased by 2.81 points (Figure 2).

## Discussion

The mean overall quality of online information for knee OA treatment was good, as measured by the modified DISCERN tool. This demonstrates a potential improvement in the quality of OA information online. A 2005 study found the quality of online OA information to be poor [15], while a 2019 study found the quality of OA information was fair [17]. This indicates that the quality of information online regarding OA, and more specifically knee OA treatment, may be improving over time. However, it is important to note that the present study utilized the modified DISCERN tool, whereas the previous studies utilized the original DISCERN tool; thus, it is difficult to directly compare results. Furthermore, total DISCERN scores for websites evaluated ranged from poor to excellent, indicating that while the mean quality was good, certain websites patients may access still lack critical information and need to continually be evaluated and improved.

The treatment information content quality for commercial and academic sites had the highest mean total DISCERN scores, which supports our hypothesis. These websites had significantly higher DISCERN scores than both nonacademic medical practice and government/patient advocacy websites. A study evaluating the quality of online information for stem cell injections for knee OA found that the DISCERN scores of academic and commercial websites were significantly greater than physician websites, which were classified as websites operated by a single physician or physician group unaffiliated with an academic institution [1]. These previous results agree with those of the present study, indicating that in general, the information found on nonacademic medical practice/private physician websites may be of lower quality compared to other sites. Since a lower DISCERN score indicates that certain information regarding treatment options is absent or only partially discussed, it is possible that the quality of nonacademic medical practice websites could improve in the future if the DISCERN tool were used as a reference of what to include in online articles.

Additionally, a study evaluated the quality of online information on Google related to COVID-19 using the DISCERN tool and the Currency, Relevance, Authority, Accuracy and Purpose test (CRAAP) [27]. Researchers found that of the five highest-quality websites, as assessed by DISCERN and CRAAP scores, the majority were commercial sites [27]. This agrees with the results of the present study, indicating that health-related information from commercial websites may be of high quality overall.

Interestingly, nonacademic medical practice websites fared the worst in all website grading measures. They had the lowest JAMA Benchmark and DISCERN scores and the highest FK grade level. Similar to the DISCERN scores, the JAMA scores of academic and commercial sites were also significantly higher than both government/patient advocacy and nonacademic medical practice websites. A study evaluating the quality of online information regarding scoliosis found that academic and physician-based websites had significantly higher JAMA Benchmark scores than other website types [28], partially agreeing with the results of our study. Additionally, there was a significant positive correlation between JAMA Benchmark and mean total DISCERN scores. A significant positive correlation between JAMA Benchmark and DISCERN scores was also found in a study evaluating the quality of online information for rotator cuff tears [16]. Taken together, these results indicate that websites with higher transparency and verifiability, as measured by the JAMA Benchmark score, often have higher-quality information as well. Thus, while the JAMA Benchmark score is not a direct measure of website content quality, a high JAMA score may be a marker of websites with high-quality information. This relationship may also help to validate the modified DISCERN tool as a measure of the quality of online information regarding treatment options.

Website readability is another important measure of a website’s quality and utility. If online health information is to serve as a useful adjunct to physician counseling, then it needs to be easily understood by most patients. Hill and Bird found a significant association between Patient Knowledge Questionnaire-OA (PKQ-OA) scores and years spent in formal education [29]. The PKQ-OA is a multiple-choice questionnaire utilized to evaluate patients’ understanding of OA [29]. This indicates that there likely exists a relationship between a patient’s education level and understanding of their disease(s) and informs us that websites whose goal is to provide health information should be easily understood by people of most education backgrounds. The recommended grade level of healthcare information to ensure appropriate patient understanding is six to eight [1]. The mean FK grade level readability of websites included in the present study was 8.3, which is at the higher end of this acceptable range. However, the FK grade level scores ranged from 3.4 to 11.8, indicating that some websites with OA treatment information may still include text that is at an inappropriately high-grade level. Interestingly, there was no significant association between website quality, as measured by the DISCERN score, and FK grade level. Overall, since the internet is widely used to access health information by people of various educational backgrounds, grade level readability is an important measure for website content creators to assess when writing and selecting information to include on their websites.

This study was not without limitations. Google’s dynamic nature makes the results of this study difficult to reproduce. As new information becomes available and websites are updated, different search results could be obtained from the same search query. Additionally, Google analyzes individual users’ search patterns and thus may generate different search results for different internet users [14]. This limitation was addressed in this study by using an incognito mode Google browser. Additionally, the list of PAA-suggested questions is different depending on which initially suggested question one clicks on. Thus, patients may obtain different search results after clicking on the PAA question that is most relevant to them. However, the questions and websites presented in this study are still representative of commonly searched questions and websites and thus provide useful insight into the quality of this online information.

## Conclusions

In conclusion, the mean overall quality of websites regarding the treatment of OA was good as evidenced by greater modified DISCERN scores. However, website quality ranged from poor to excellent, indicating that some websites are still missing key information patients may find useful when assessing treatment options online. There was a significant positive correlation between total DISCERN and JAMA Benchmark scores, highlighting that website with greater transparency also often had high quality information. Nonacademic medical practice websites had the worst JAMA Benchmark, DISCERN, and FK grade level readability scores, indicating a need for quality improvement of these websites. Since nonacademic medical practice sites may be directly or indirectly managed by physicians and/or orthopedic surgeons, identifying these shortcomings may provide an opportunity for surgeons to improve patient education by improving the quality of their websites. Additionally, because this study showed that the quality, transparency and readability of websites varies greatly, it may be useful for physicians to provide patients with high-quality online resources they can use when looking for more information on treatments and OA in general.
